# How effective is the addition of specific exercise therapy for patients after anterior cruciate ligament surgery? A systematic review and meta-analysis

**DOI:** 10.3389/fphys.2025.1501458

**Published:** 2025-01-24

**Authors:** Hao Zhou, Jia Qian, Yu-Mei Xing, Long Cui, Yi-Feng Bu

**Affiliations:** ^1^ Physical Education Institute, Jiangsu Normal University, Xuzhou, China; ^2^ Library, Jiangsu Normal University, Xuzhou, China

**Keywords:** exercise therapy, anterior cruciate ligament reconstruction, anterior cruciate ligament injuries, kinesiotherapy, anterior cruciate ligament

## Abstract

**Context:**

Anterior cruciate ligament (ACL) injuries are prevalent in sports and often require surgical intervention followed by rehabilitation. Several rehabilitation methods have been used for patients after ACL surgery.

**Objective:**

This study aimed to assess the overall efficacy of exercise therapy in improving outcomes for patients following ACL surgery using a systematic review and meta-analysis of randomized controlled trials (RCTs).

**Data sources:**

PubMed, Web of Science, Embase, and the Cochrane Library were searched for randomized controlled trials published from 1 January 2000 to 30 August 2024. Study quality was assessed using the Cochrane Risk-of-Bias tool.

**Study selection:**

A total of 11 randomized controlled trials (whole-body vibration training = 4, core-stability training = 2, strength training = 3, blood flow restriction training = 1, and aquatic training = 1) involving 552 anterior cruciate ligament surgery patients were included.

**Data extraction:**

Two researchers individually screened the key information for each eligible study and evaluated the quality of the studies. Any dispute was discussed by a third researcher.

**Results:**

Compared with conventional therapy, exercise therapy significantly reduced pain scores (mean difference: −0.53, 95% CI: −0.82 to −0.24, and *p* < 0.001) and improved muscle strength (flexion: 13.76 and extension: 12.46) and knee function (effect size: 2.06 and *p* = 0.001). Secondary outcomes, although less pronounced, also demonstrated improvement.

**Limitation:**

The sources of heterogeneity among the included studies were not fully identified, particularly concerning variations in exercise protocols or patient characteristics. Additionally, the therapeutic effects of specific exercise modalities (e.g., strength training versus aquatic training) were not directly compared.

**Conclusion:**

Exercise therapy is effective in reducing pain, enhancing muscle strength, and improving knee function in ACL surgery patients. These findings underscore the importance of integrating tailored exercise therapies into rehabilitation programs.

**Systematic review registration number:**

https://www.crd.york.ac.uk/PROSPERO/, identifier registration number. CRD42023476653.

## Introduction

The anterior cruciate ligament (ACL), an intra-articular ligament located outside the synovial membrane (Diermeier et al., 2020), plays as a pivotal role in the knee joint. It originates from the medial wall of the lateral condyle of the femur and consists of the anteromedial bundle and posterolateral bundle (Samuelsson et al., 2009; Acevedo et al., 2014). The ACL primarily acts as a stabilizer for the knee joint by resisting hyperextension of the knee, anterior tibia, and rotation (Acevedo et al., 2014). Additionally, it is also an important proprioceptive receptor that can signal changes in the knee joint (van Melick et al., 2016). However, many risk factors may cause damage to the ACL. The internal factors include high body mass index, lower limb muscle strength deficiency, sex (women are at greater risk of injury), and the state of the body during exercise (Diermeier et al., 2020; Acevedo et al., 2014). The external factors encompass the sports venue, weather, and competition level (Acevedo et al., 2014). At the same time, ACL is relatively fragile in response to acute torsional or shear forces (Jenkins et al., 2022). Hence, ACL injuries often occur during sudden sports activities.

ACL injury represents approximately 50% of knee injuries, of which 70%–80% were non-contact injuries (Kaeding et al., 2017; Boden et al., 2000). Non-contact injuries usually occur during a jump landing, swerving, or stopping (Boden et al., 2000). Once the ACL is injured, the tibia’s forward motion relative to the femur cannot be confined, thereby impairing the support, flexion, and extension functions of the lower limbs. In a short period of time, a large amount of fluid accumulates in the knee joint, and the degree of motion decreases significantly (Jenkins et al., 2022). A literature review in 2022 found that there were approximately 80,000 to 120,000 patients with ACL injuries in the United States each year (Gornitzky et al., 2016). After the injury occurred, some patients who were reluctant to undergo surgery also achieved better results. However, for young people and those who wish to return to pre-injury activity levels, surgery is the only option (Gerber et al., 2009). Bone–patella tendon–bone (BPTB) graft is considered the gold standard for surgical reconstruction (Diermeier et al., 2020), but it increases the risk of patellofemoral osteoarthritis (OA) and secondary diseases (Papageorgiou et al., 2001; Canzone et al., 2024). Compared to the BPTB graft, the autologous hamstring tendon (HT) graft reduces the risk of secondary diseases, but the rotational relaxation of the grafted ligaments increases over time (Samuelsson et al., 2009). Therefore, rehabilitation after surgery is equally important.

For patients undergoing ACL surgery (ACLS), rehabilitation is to restore joint movement, lower-limb muscle mass, and knee function (Jenkins et al., 2022) so as to achieve pre-injury activity levels and functional status. Clinical guidelines from the American Physical Therapy Association state that rehabilitation can enhance lower-limb muscle strength (Logerstedt et al., 2010). At the same time, it is recommended to conduct an early joint range of motion association 1 week after surgery to prevent the occurrence of soft tissue diseases such as joint contracture (Logerstedt et al., 2010). In addition, a study on postoperative rehabilitation has found that these training strategies can significantly reduce the risk of secondary injuries such as meniscus and arthritis (Suter and Herzog, 2000).

However, a standard postoperative rehabilitation strategy is lacking, and the design (Jenkins et al., 2022), type, and timing of rehabilitation programs vary widely (Kotsifaki et al., 2023; Buckthorpe, 2021). Therefore, it is particularly important to elucidate the advantages and disadvantages of various rehabilitation programs. Traditional therapy is currently the mainstay rehabilitation approach in clinical practice, which can facilitate the recovery of postoperative muscle strength and motion (Capin et al., 2018; Clark et al., 2015). Usually, it uses physical instrument therapy (e.g., cryotherapy and neuromuscular electrical stimulation), physical therapist-assisted therapy (e.g., continuous passive motion and Kinesio-taping), and some common forms of exercise (most are simple strength training) to achieve the effect of rehabilitation (Kotsifaki et al., 2023). However, due to the lack of targeted exercise training or treatment, patient recovery and outcomes are often compromised. Therefore, it is necessary to include specific exercises in the patient’s rehabilitation program.

Exercise therapy (ET) techniques mainly include strength training, progressive training, and functional training (Kotsifaki et al., 2023; Korakakis et al., 2021; Beard and Dodd, 1998; Culvenor et al., 2022), which are essential for patients to regain their original mobility. One of the most common training methods in exercise therapy, strength training, commonly used in the postoperative rehabilitation of sport-related injuries, is considered the best strategy to prevent muscle atrophy (Logerstedt et al., 2010; Kotsifaki et al., 2023; Buckthorpe, 2021). In addition to the conventional exercise training methods, some new training methods have emerged in other fields. As for postoperative rehabilitation training for ACL patients, these new methods are also worth studying. For example, whole-body vibration training (WBVT) can improve neuromuscular activation to some extent (Moezy et al., 2008). It works by providing rapid, brief, and repetitive intermittent vibrations to the body, causing the body to produce vertical, horizontal, or interactive displacement (Shantakumari and Ahmed, 2023). The trainer himself needs to use body force to maintain balance in order to achieve the purpose of training (Shantakumari and Ahmed, 2023). This approach is more effective in enhancing postural control, muscle, and stability in patients after ACLS than traditional rehabilitation methods (Fu et al., 2013). Moreover, some new training methods have emerged in recent years, such as blood flow restriction training (BFRT) and water-based exercise, which have been used for the rehabilitation of patients after ACLS. The mechanism of BFRT involves the trainer creating an ischemic and hypoxic environment in the affected area during BFR, which increases metabolism (Hughes et al., 2017). At the same time, the combination of BFR and exercise can induce mechanical tension (Hughes et al., 2017). These are key factors in the development of muscle hypertrophy (Hughes et al., 2017; Pearson and Hussain, 2015). Current research on BFRT has focused on the mechanism of delaying muscle atrophy (Charles et al., 2020). Water-based exercise involves performing exercises, which are generally performed on land, in water. This method is characterized by exercising in water. On one hand, due to the presence of buoyancy, the risk of injury is reduced for individuals with excessive weight or reduced lower-limb strength (Zhu et al., 2023). On the other hand, due to the presence of resistance in water, exercisers will be subjected to additional stimulation (Zhu et al., 2023).

In recent years, published reviews of the efficacy of ET in patients after ACLS have mostly focused on a single exercise or just an evidence-based guideline (Kotsifaki et al., 2023; Buckthorpe, 2021; Culvenor et al., 2022; Poretti et al., 2023; Wengle et al., 2022). Meanwhile, clinical trials have high specificity, but few articles have systematically reviewed the role of exercise therapy in rehabilitation programs. To address these shortcomings, we conducted a systematic review and meta-analysis of randomized controlled trials (RCTs) published since 2000 to explore the role of exercise therapy in the rehabilitation of patients after ACLS.

## Methods

### Procedures

This study protocol was registered in the PROSPERO database (registration no. CRD42023476653; 2023.11.23), and this study was reported according to the PRISMA guidelines (Moher et al., 2009).

### Search strategy

PubMed, Web of Science, Embase, and the Cochrane Library were searched for RCTs on ET for the rehabilitation of patients after ACLS. Mesh terms and free-text words were combined to create the search terms, including “anterior cruciate ligament reconstruction,” “anterior cruciate ligament injuries,” “exercise therapy,” “kinesiotherapy,” and “random allocation.” The complete search strategy is detailed in Supplementary Material S1. Two researchers independently searched for potential studies published between 1 January 2000 and 30 August 2024 (we only included studies published within this period). Disagreements, if any, were addressed by a third reviewer.

### Eligibility criteria

The criteria for inclusion were designed following the PICOS principle, which was as follows (Diermeier et al., 2020): participants: patients had to undergo ACLS (Samuelsson et al., 2009). Intervention: the intervention group used ET, which differed from the one used by the control group (strength training, aquatic training, and vibration training) (Acevedo et al., 2014). Control: the control group used conventional rehabilitation (van Melick et al., 2016). Study design: RCTs published in English between 2000 and 2024 were eligible (Jenkins et al., 2022). Only peer-reviewed and English-language articles were eligible. The exclusion criteria were as follows (Diermeier et al., 2020): patients with ACL injury who did not undergo surgery (Samuelsson et al., 2009); ET types that were not significantly different from that of the control group (such as aerobic exercise, cycling, or walking; these exercises were used in the usual treatment group and could not be compared to the control group in some studies) or reported outcome measures (psychological indexes or electroencephalogram indexes; these results were relevant to ACL rehabilitation, but our study focused on outcomes of joint function rather than psychological outcomes) that were unrelated (Acevedo et al., 2014); a sample size of fewer than 10 cases in each group (if the sample size was too small, we believe that the quality of the results was low, and there may be particularities and chance involved) (van Melick et al., 2016); case reports, animal experiments, review articles, and unpublished articles (Jenkins et al., 2022); the control group used the same or other exercise rehabilitation interventions as the experimental group (Kaeding et al., 2017); and research methods were of low quality, or peer review was unclear. Details are shown in Table 1.

**TABLE 1:** PICOS.

PICOS	Content of this study
Participants	Patients who received anterior cruciate ligament surgery
Intervention	The intervention group used specific exercise therapy, which differed from the control group This article included quadriceps exercises, whole-body vibration training, core-stability training, aquatic training, blood flow restriction training, isokinetic muscle strength training, and Nordic hamstring training
Control	Conventional rehabilitation It is composed of a variety of rehabilitation ingredients, and the most common items are as follows (Culvenor et al., 2022; Kruse et al., 2012): 1. Physical therapy (physiotherapist works with the aid of an instrument) 2. Brace-assisted therapy 3. Drug therapy, such as placebo and vitamin 4. Progressive strength exercises 5. Progressive range of motion exercises 6. Psychological therapy
Outcomes	Outcomes related to pain, muscle strength, and joint function were the primary outcome measures (this article included pain, isokinetic muscle strength, and knee joint function score) Outcomes related to knee balance function and proprioception were used as the secondary outcome indicators (this article included the knee joint reposition test and postural stability test)
Study design	RCTs published in English between 2000 and 2024 (only peer-reviewed and English-language articles were eligible)

### Data extraction

After removing duplicates, unrelated studies were filtered out through the examination of titles and abstracts. The full texts of the remaining studies were carefully read to screen eligible trials. Two reviewers independently undertook data extraction. The extracted data encompassed the author, information on the participants (such as origin, number, type of operation, and age), and intervention design (such as inserted exercise type, details of exercise, and duration). We used Microsoft Excel to manage and convert data. All results were extracted in the form of mean and standard deviation, and only two decimal places were retained. In the event of data loss, we contacted the authors to obtain the original data. If the authors could not be contacted, this part of the data was excluded as missing data. Any disagreements were addressed by a third reviewer. The three researchers discussed the dissents and finally reached a consensus.

### Outcome measures

The outcome measures were recorded at each assessment point (baseline and post-intervention). The primary outcomes were pain, the isokinetic muscle strength test, and the knee function score. Among them, pain was measured by the visual analog scale (VAS), one of the prevalent methods to assess the health-associated quality of life (Åström et al., 2023). The isokinetic muscle strength test is a safe method to measure muscle strength using a specific instrument and a fixed angular speed (Cheng et al., 2019). The scales for measuring knee joint function were different across studies, and we finally analyzed the Lysholm and International Knee Documentation Committee (IKDC) scores. Secondary outcomes encompassed the knee joint reduction test and postural stability test. In addition, these outcomes were depicted as the mean and standard deviation. In case of disagreements, a third reviewer was consulted to help reach a resolution.

### Risk of bias

Two reviewers, working independently, evaluated the risk of bias (RoB) in the recruited RCTs utilizing the Cochrane Risk-of-Bias Tool (RoB 2.0). Each study was assessed and graded as “low RoB,” “some concerns,” or “high RoB” in five domains, namely, randomization process, deviations from intended intervention, missing outcome data, outcome measurement, and selection of the reported results, including deviations from the registered protocol. In cases where a trial exhibited a high risk in at least one domain, it was graded as having an overall high RoB, while it was graded as having an overall low risk if the RoB across all domains was low.

### Data analysis

The first step was to calculate the changes in outcomes of the experimental and control groups from baseline to post-intervention. The mean difference (MD) or weighted mean difference (WMD) with a 95% confidence interval (CI) was reported for continuous data. STATA 15.0 was used for meta-analysis. Cochran’s Q test and Higgins I^2^ test were executed to gauge heterogeneity. *p* < 0.10 or I^2^ > 50% implied substantial heterogeneity, and then a random-effects model was adopted. Otherwise, a fixed-effects model was utilized. Sensitivity and subgroup analyses were conducted to determine the origin of heterogeneity in cases of high heterogeneity. *p* < 0.05 indicated a statistical difference. To assess publication bias statistically, Egger’s and Begg’s tests were employed, and funnel plots were used for visual inspection. The trim and filling method was utilized to clarify the influence of publication bias on the meta-analysis result.

## Result

### Search result

**FIGURE 1:**
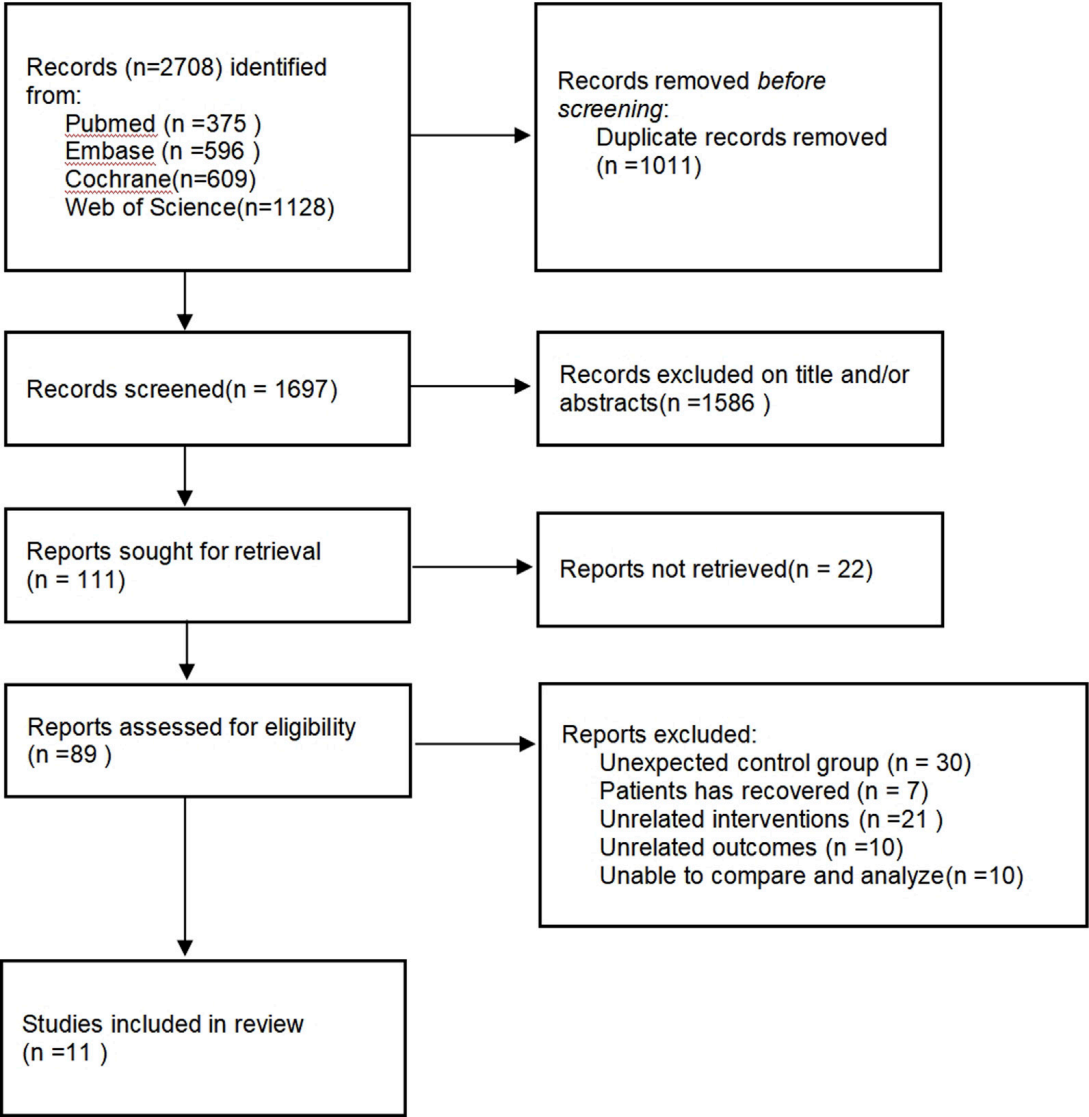
Flow chart of study selection.

Figure 1 displays the literature retrieval and screening process. A total of 2,708 potential studies were obtained from electronic databases. Based on the title, abstract, and duplicate records, 2,597 studies were eliminated. After reading the full texts of the remaining 111 reports, 100 were ruled out due to unexpected control groups (n = 30), unrelated interventions (n = 21), and unrelated outcomes (n = 10). Finally, 11 RCTs were included.

### Study characteristics

The included RCTs were published between 2000 and 2023, and the exercise intervention, duration, and outcome measures varied across these studies. There were 552 participants from seven regions, and all participants underwent ACL surgery recently. The sample size of each group ranged from 10 to 55 cases, with participants aged 10–36 years, and most participants were male. The treatment duration varied from 1 to 6 months. WBVT was used in four studies, core-stability training in two studies, strength training in three studies, BFRT in one study, and aquatic training in one study. The detailed characteristics are provided in Tables 2, 3.

**TABLE 2:** Characteristics of study design, intervention, and participants.

No.	Author	Year	Country	Source	Object features	Sample size (male)	Age, mean ± SD	Type of operation	Experimental group inserted exercise therapy	Exercise intervention starting point	Overall treatment length
1	Shaw	2005	South Australia	Monocentric	Generation popular^a^	G1 = 48 (34)	G1 = 18.4 ± 8.1	Bone–patellar tendon–bone or semitendinosus-hamstring graft	Early quadriceps exercises	Start immediately after surgery	Six months
						G2 = 55 (41)	G2 = 28.8 ± 9.3				
2	Moezy	2008	Iran	Multicenter	Professional athletes	G1 = 10 (10)	G1 = 24.51 ± 3.38	Central bone–patellar tendon approach	Whole-body vibration training	Start immediately after surgery	one month
						G2 = 10 (10)	G2 = 22.7 ± 3.77				
3	Fu	2013	China, Hong Kong	Monocentric	Generation popular	G1 = 24 (18)	G1 = 23.3 ± 5.2	Hamstring tendon reconstruction	Whole-body vibration training	One month after surgery	Six months
						G2 = 24 (14)	G2 = 25.2 ± 7.3				
4	Pistone	2016	Italy	Monocentric	Generation popular	G1 = 17	G1 = 29 ± 7	Semitendinosus graft	Whole-body vibration training	One month after surgery	Three months
						G2 = 17	G2 = 27 ± 7				
5	Priyanka	2017	India	—	Generation popular	G1 = 30 (27)	G1 = 29 ± 5.5	Hamstring tendon graft	Core-stability training	—	Four weeks
						G2 = 30 (27)	G2 = 29 ± 5.3				
6	Costantino	2018	Italy	Monocentric	Professional athletes	G1 = 19 (0)	G1 = 25.47 ± 2.01	Patellar tendon graft	Whole-body vibration training	Thirteen weeks after surgery	Six months
						G2 = 19 (0)	G2 = 25.42 ± 2.39				
7	Li	2019	China	Monocentric	Generation popular	G1 = 37 (37)	G1 = 26.5 ± 3.1	Hamstring tendon graft	Core-stability training	One month after surgery	Six months
						G2 = 37 (37)	G2 = 27.6 ± 2.4				
8	Hajouj	2021	Iran	Monocentric	Professional or amateur athletes	G1 = 19 (19)	G1 = 23.79 ± 3.04	Hamstring tendon graft	Aquatic training	Seven weeks after surgery	Thirteen weeks
						G2 = 19 (19)	G2 = 24.68 ± 3.78				
9	Khalil	2023	Egypt	—	Generation popular	G1 = 18 (15)	G1 = 23.78 ± 3.934	Semitendinosus muscle tendon graft	Blood flow restriction training	One week after surgery	Three months
						G2 = 18 (16)	G2 = 25.22 ± 4.76				
10	Wang	2023	China	—	Professional athletes	G1 = 21	G1 = 21.6 ± 3.2	Bone–patellar tendon–bone reconstruction	Isokinetic muscle strength training	Four weeks after surgery	Two months
						G2 = 20	G2 = 22.2 ± 2.8				
11	Chen	2023	China	—	Generation popular	G1 = 30 (Moher et al., 2009)	G1 = 23.78 ± 3.934	Hamstring tendon graft	Nordic hamstring training	Three weeks after surgery	Twenty-four weeks
						G2 = 30 (Moher et al., 2009)	G2 = 25.22 ± 4.76				

G1, group1 (exercise therapy group); G2, group2 (conventional group).

^a^ refers to general hospital patients with no reported exercise experience.

**TABLE 3:** Details of intervention and outcomes.

No.	Exercise intervention details	Outcomes and measurement
1	1. Foot and ankle exercises 2. Active, assisted knee flexion 3. Calf stretches 4. Passive knee extension 5. Standing posture 6. Gait education 7. Passive knee extension with weight 8. Static quadriceps contraction (10 repetitions, three times daily) 9. Straight leg raises (10 repetitions, three times daily)	1. Range of motion tests, active and passive 2. For pain, the VAS scale was used during exercise and rest 3. For the functional hop test, single and triple hop tests were used 4. The Cincinnati Knee Rating System was used to systematically evaluate knee joint 5. For the isokinetic muscle strength test, an isokinetic instrument was used to test at the set angular speed (60 deg/sec) 6. For the knee laxity test, KT-1000 was used to test knee joint relaxation in two measurements (15 lb and 20 lb)
2	Patients performed nine movement exercises on a whole-body vibrator^a^ 1. Static position, standing with knees slightly bent, feet in the middle of platform and slightly apart, back straight 2. Static position, one-leg stance with knee slightly bent, foot in the middle of platform, back straight 3. Static or dynamic position, mini squat 4. Static or dynamic position, one-leg mini squat 5. Static or dynamic position, deep squat 6. Static or dynamic position, one-leg deep squat 7. Static or dynamic position, wide stance squat 8. Static or dynamic position, lunge, one foot in the middle of platform, back straight, bend the knee approximately 90° 9. Static or dynamic position, toe standing	1. For the postural stability test, the Biodex Stability System was used to measure overall, anterior–posterior and medial–lateral stability indices (OSI, APSI, and MLSI, respectively) 2. For the knee joint reposition test, the Biodex dynamometer system was used to measure the angle of knee movement and see whether the required angle (30° and 60°) is achieved
3	Patients performed 11 movement exercises on a whole-body vibrator^b^ 1. Basic position, standing with force evenly distributed in the middle of the exercise platform, feet in shoulder width symmetrically, knees slightly bent and back straight, press a ball between the knees 2. Start with basic position, weight shifting to forefoot with heels off, alternate the height of heels from ground simultaneously 3. Start with basic position, weight shifting to heels with forefoot off ground, alternate the height of toes from ground simultaneously 4. Mini squat, same as basic position, but with knees flexion at approximately 45° 5. Deep squat, same as basic position, but with knees flexion at approximately 90° 6. Wide stance squat, symmetrical and stable foot position with wide base, knees bent with back straight, slowly up and down, 5 s up and 5 s down 7. Lunges, one foot in the middle of platform, back straight, knee flexion 90°, another foot on ground, weight shifting to the foot placed on the platform, repeat for another foot 8. Single-leg stance, single-leg standing alternatively with knee slightly bent, foot in the middle of platform and back straight, free leg held up in a relaxed manner 9. Sit on the platform with big toes pointing upward and back straight 10. Place your calves on the platform and relax 11. Place your medial thigh on the platform and relax	1. For the joint position sense test, the Biodex dynamometer system was used to measure the angle of knee movement and see whether the required angle (30° and 60°) is achieved 2. For the postural control test, the Biodex Stability System was used to measure the overall stability index (OSI), anterior–posterior stability index (API), and medial–lateral stability index (MLI) 3. For the isokinetic test, Cybex NORM was used to test at the set angular speed (60, 180, and 300 deg/sec) 4. For the functional test, single-legged and triple hop tests were used 5. For the knee laxity test, KT-1000 was used to test knee joint relaxation
4	Patients began whole-body vibration (WBV) at week 4, and the protocol was as follows: 1. At week 4, the participants started undergoing three series of 1-min WBV bouts at the optimal vibration frequency (OVF), with 1-min resting between the series 2. At week 5, five series of 1-min WBV bouts at the OVF, with 1-min resting between the series 3. At week 6, seven series of 1-min WBV bouts at the OVF, with 1-min resting between the series 4. At week 7, 10 series of 1-min WBV bouts at the OVF, with 1-min resting between the series	1. The strength test was used to test for the maximal isometric voluntary contraction of the knee extensor and flexor muscles in each limb 2. For the balance test, a pressure platform was used to quantify the displacement of the feet center of foot pressure 3. Knee joint function was measured using the Lysholm score
5	1. Level 1, core activation, draw in and hold 10 s 2. Level 2, opposite lower extremity on mat, bent leg fall out 3. Level 3, opposite lower extremity on a table 4. Level 4, hold opposite lower extremity at 90 degrees of hip flexion with upper extremity 5. Level 5, hold opposite lower extremity at 90 degrees of hip flexion without upper extremity 6. Level 6, bilateral lower extremity movement (Level 3, 4, 5, and 6 action options include lift leg to 90° hip flexion, slide heel to extend knee, and lift straight leg to 45°)	1. For assessing pain, the VAS scale was used 2. In terms of the range of motion tests, the full range of motion of the patient’s knee joint was measured 3. For knee joint function, the modified Lysholm scoring scale (MLSS) and Tegner activity level (TAL) were used
6	Participants perform nine movement exercises on a whole-body vibrator 1. Quarter squatting, the subjects were requested to remain standing upright on the platform with knees slightly bent to approximately 25° 2. Quarter squatting on one leg (operated limb) (Eight weeks duration, three sessions per week, support on one foot and two feet, one set of six repetitions for each position, each repetition lasting 1 minute, 1 minute rest period between each repetition, and 2 minutes rest period between each set)	1. For the isokinetic muscle strength test, an isokinetic instrument was used to test knee flexion and extension peak torque, strength, and endurance
7	1. Transversus abdominis and multifidus muscle contraction exercises, patients in the supine position inhaled deeply to relax the abdominal muscles and exhaled slowly to contract the abdomen as much as possible for 10 s, repeat 20 times 2. Body trunk control training, patients in the supine position extended the scapula while moving abdominal muscles and bending the knee for oblique abdominal exercises, repeat 20 times	1. Gait correlation result, stride frequency, stride length, and gait period were measured 2. For the range of motion tests, the full range of motion of the patient’s knee joint was measured 3. Joint peak reaction force, test hip, knee, and ankle peak reaction force 4. Knee joint function was measured using the Lysholm score
8	Patients underwent two weekly 45–60 min sessions of innovative proprioception aquatic exercises 1. Single-leg stance with eyes open 2. Single-leg stance with eyes closed 3. Single-leg stance with leg swing and eyes open 4. Single-leg stance with leg swing and eyes closed 5. Single-leg squat with eyes open and knee flexed at 30° 6. Single-leg squat with eyes closed and knee flexed at 30° 7. Double-leg stance on foam roll, subject in standing position, barefoot, double-legged support 8. Single-leg stance on foam roll, subject in standing position, barefoot, one-legged support 9. Single-leg stance with leg swing on foam roll, subject in standing position, barefoot, one-legged support 10. Rollover walking forward with crossed arms on foam roll, subject in standing position on foam roll, barefoot, double-leg support, walking forward and arms crossed 11. Single-leg stance on foam roll and throwing the ball subject in a standing position on foam roll, one-legged support, throwing the ball to the subject and throwing it back to the therapist 12. Rollover walking forward on foam roll and throwing the ball, subject in standing position on foam roll, barefoot, double-leg support, walking forward, throwing the ball to the subject and throwing it back to the therapist	1. Proprioception assessment 2. For assessing pain, the VAS scale was used 3. For assessing knee function, the IKDC questionnaire was used
9	Blood flow restriction of 80% limb occlusive pressure was added to neuromuscular electrical stimulation (NMES) and mentioned strength training 1. Continue NMES 10 min 2. Stretching exercise to calf muscle, 15 s, three repetitions 3. Single-leg stance supported 30–60 s 4. Stationary bike level 2 or 3 on bike, 10–15 min 5. Half Swiss ball weight shifting, 30 repetitions 6. Single-leg standing open and closed eyes, 30–60 s 7. Static mini-squat with co-contraction, 30 s, three repetitions	1. For pain assessment, the VAS scale was used
10	Isokinetic testing apparatus was used to perform the isokinetic training of flexion and extension at 60°/s and 240°/s (five sets of 12 repetitions/set with 1 min rest between sets and 5 min between angles)	1. For the isokinetic muscle strength test, an isokinetic instrument was used to test at the set angular speed (60° and 240°/s) 2. For the proprioception test, a knee kinesthetic device was used with a reliability of 0.93 3. For the balance ability test, a 3D measuring bench was used to test the average speed of the pressure center in the anterior–posterior (AP) direction and average speed of the pressure center in the medial–lateral (ML) direction
11	The patient was instructed to kneel on both knees, keep the torso upright, place the hands on either side of the torso and tense the body; the therapist secured the patient’s ankles in place and applied pressure to ensure that the patient’s tibia remained in contact with the mat throughout the movement; the patient’s torso slowly descended forward while slowing down the descent of the body through the centrifugal action of the popliteal muscle group to resist the forward descending movement until it landed flat on the mat	1. For the muscle stress test, an isokinetic instrument was used to test at the set angular speed (60° and 120°/s) 2. For knee examination, Lachmen’s test, the anterior drawer test, and KT-1000 were used to measure ligament strength

^a^ With the passage of training time, the duration of each group was lengthened (30–60 s), the vibration frequency was increased (30–50 Hz), the rest between groups was reduced (60–30 s), and the static exercise was changed from static exercise to static and dynamic exercise.

^b^ With the passage of training time, the duration of each group was lengthened (30–40 s), the vibration frequency was increased (35–50 Hz), and the rest between groups was reduced (30–15 s).

### RoB

**FIGURE 2:**
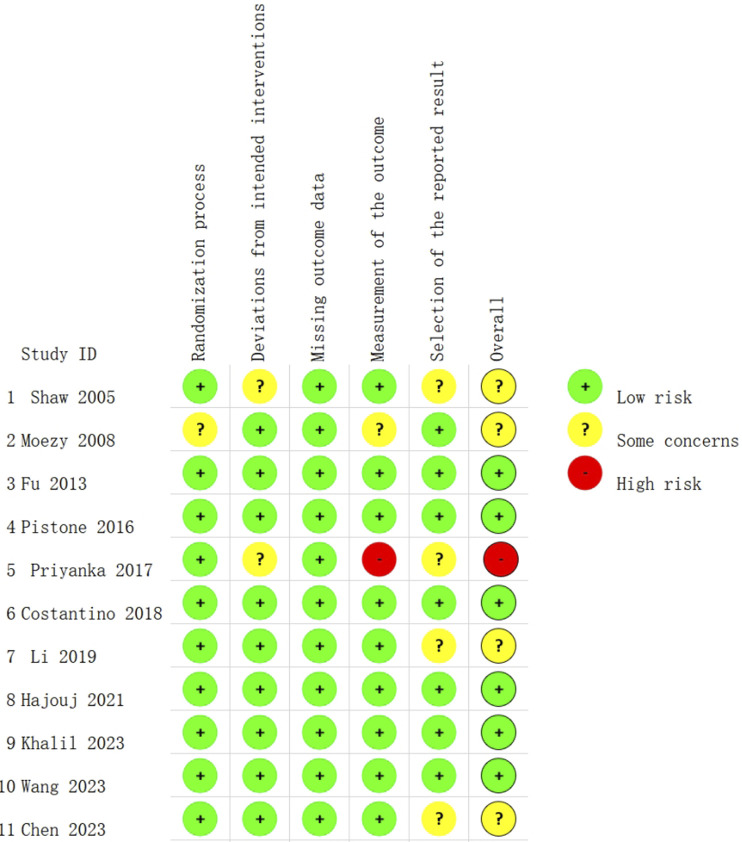
Risk of bias summary.

The Cochrane Risk-of-Bias Tool (RoB 2.0) was used to evaluate the RoB. Figure 2 shows the RoB assessment results. Each RCT described the random sequence generation process. In terms of outcome reporting, four studies (Shaw et al., 2005; Priyanka et al., 2017; Li and Xie, 2019; Chen et al., 2023) were deemed to have some concerns. The deviation from the intended intervention was not clear in two studies (Shaw et al., 2005; Priyanka et al., 2017), and no studies had a RoB of missing outcome data. Overall, six studies (Fu et al., 2013; Pistone et al., 2016; Costantino et al., 2018; Hajouj et al., 2021; Wang et al., 2023; Khalil et al., 2023) were graded as having low RoB, four studies (Moezy et al., 2008; Shaw et al., 2005; Li and Xie, 2019; Chen et al., 2023) were graded as having some concern, and only one study (Priyanka et al., 2017) was graded as having high RoB. The high RoB may be ascribed to the insufficient description of relevant information on participants.

### Effect of ET on primary outcomes

#### Pain rated by VAS

**FIGURE 3:**
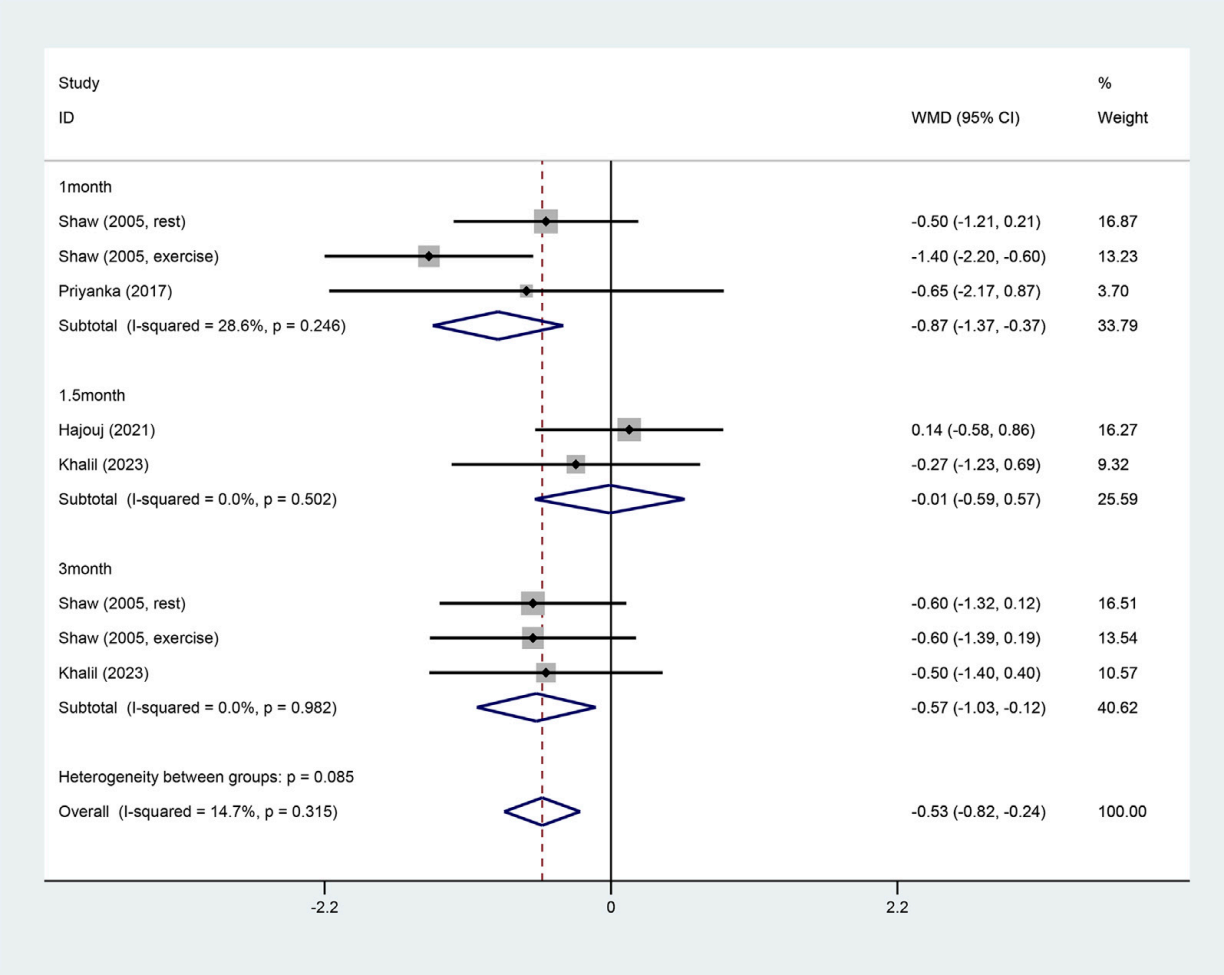
Pain forest map.

Four studies reported pain (Shaw et al., 2005; Priyanka et al., 2017; Hajouj et al., 2021; Khalil et al., 2023). Due to small heterogeneity (I^2^ = 14.7%), a fixed-effect model was adopted. ET significantly lowered VAS scores compared to conventional rehabilitation (WMD = −0.53, 95% CI: −0.82 ‐ −0.24, and *p* < 0.001). Additionally, a subgroup analysis by intervention duration found that ET significantly relieved pain in both short term (WMD = −0.87, 95% CI: −1.37 ‐ −0.37, and *p =* 0.001) and long term (WMD = −0.57, 95% CI: −1.03 ‐ −0.12, and *p* = 0.014) but not in the medium term (WMD = −0.01, 95% CI: −0.59 ‐ 0.57, and *p* = 0.975) (Figure 3).

#### Isokinetic muscle strength

**FIGURE 4:**
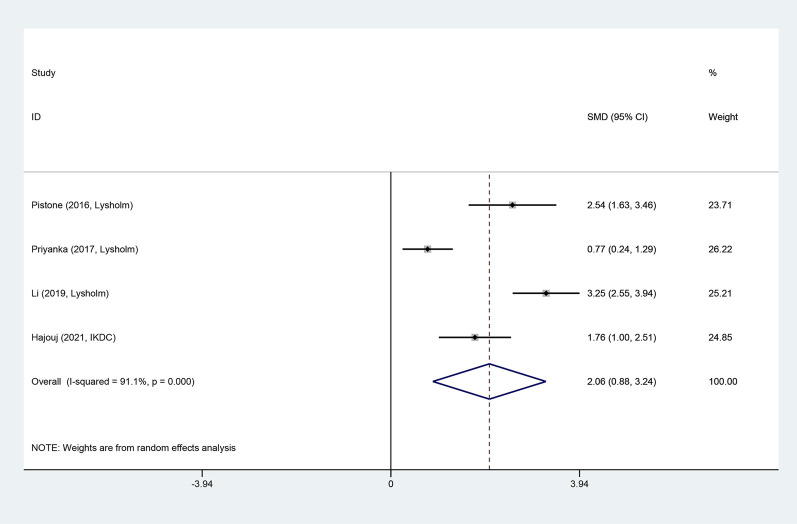
Isokinetic flexion forest map.

**FIGURE 5:**
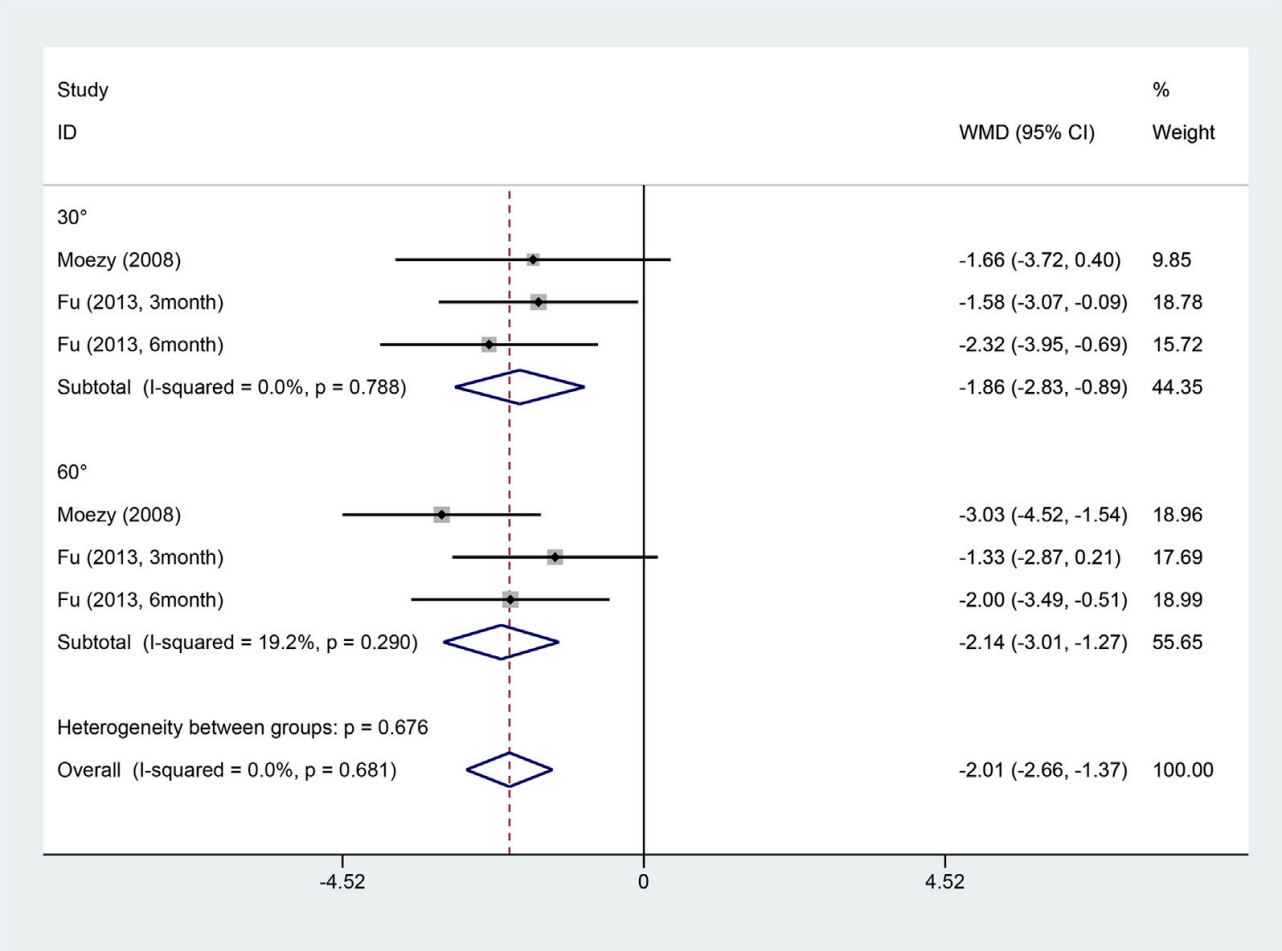
Isokinetic extension forest map.

Four articles reported isokinetic muscle strength (Fu et al., 2013; Chen et al., 2023; Costantino et al., 2018; Wang et al., 2023). Due to large heterogeneity (flexion I^2^ = 63% and extension I^2^ = 88.9%), a random-effects model was employed. ET significantly improved isokinetic muscle strength at both flexion (WMD = 13.76, 95% CI: 11.32 ‐ 16.21, and *p <* 0.001) and extension (WMD = 12.46, 95% CI: 7.35 ‐ 17.56, and *p <* 0.001). A subgroup analysis by angular velocity found significant differences between ET and conventional rehabilitation (Figures 4, 5).

#### Knee joint function score

**FIGURE 6:**
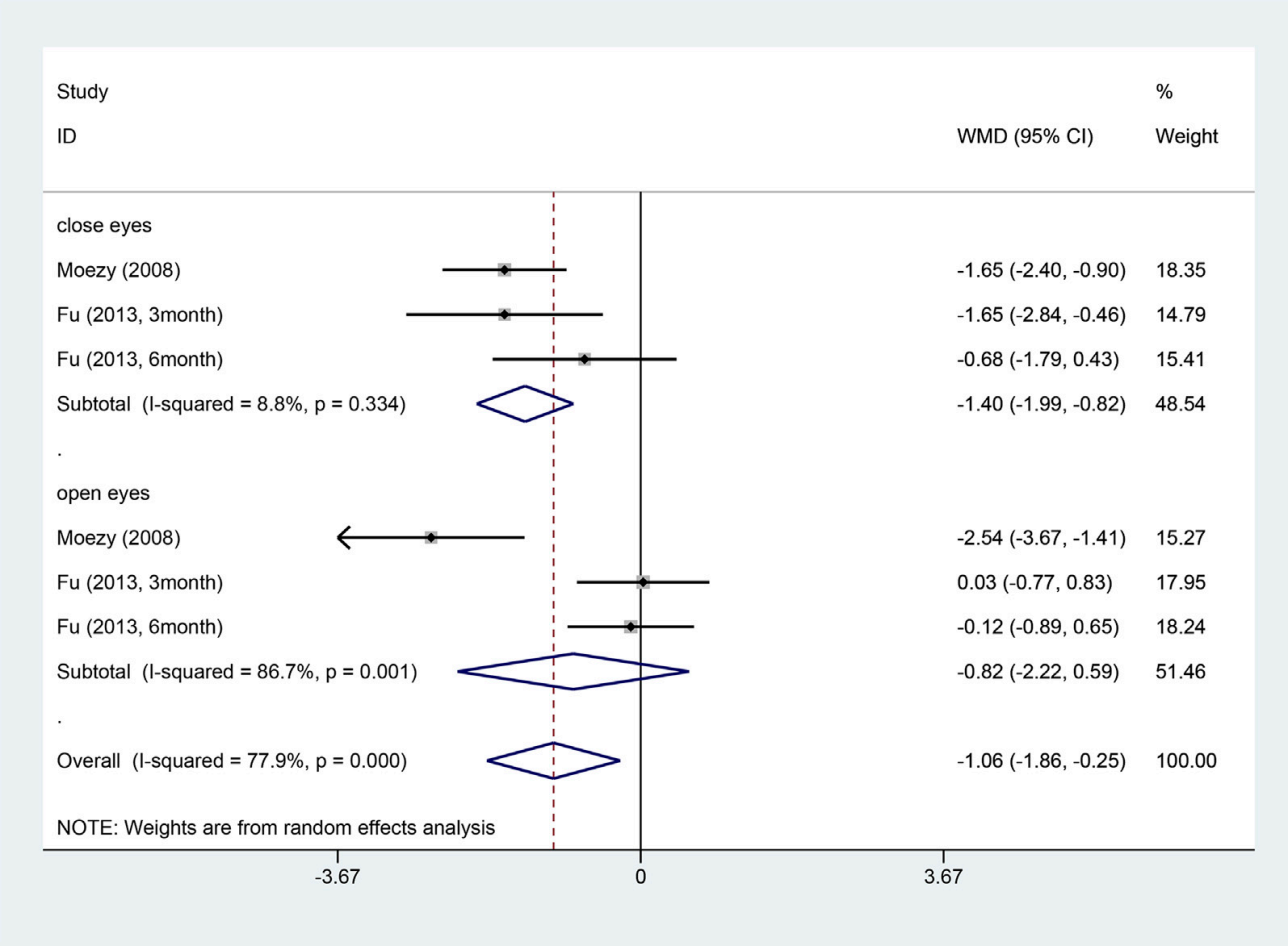
Knee joint function score forest map.

Four articles reported knee joint function scores after the intervention. Due to large heterogeneity (I^2^ = 91.1%), a random-effects model was utilized. ET significantly improved knee joint function (SMD = 2.06, 95% CI: 0.88 ‐ 3.24, and *p =* 0.001). In the original studies, although both the ET and conventional rehabilitation groups had an average knee function score of more than 70 points (a score of over 65 indicated a favorable knee function), the ET group exhibited higher scores than the conventional rehabilitation group (Figure 6).

### Effect of ET on secondary outcomes

**FIGURE 7:**
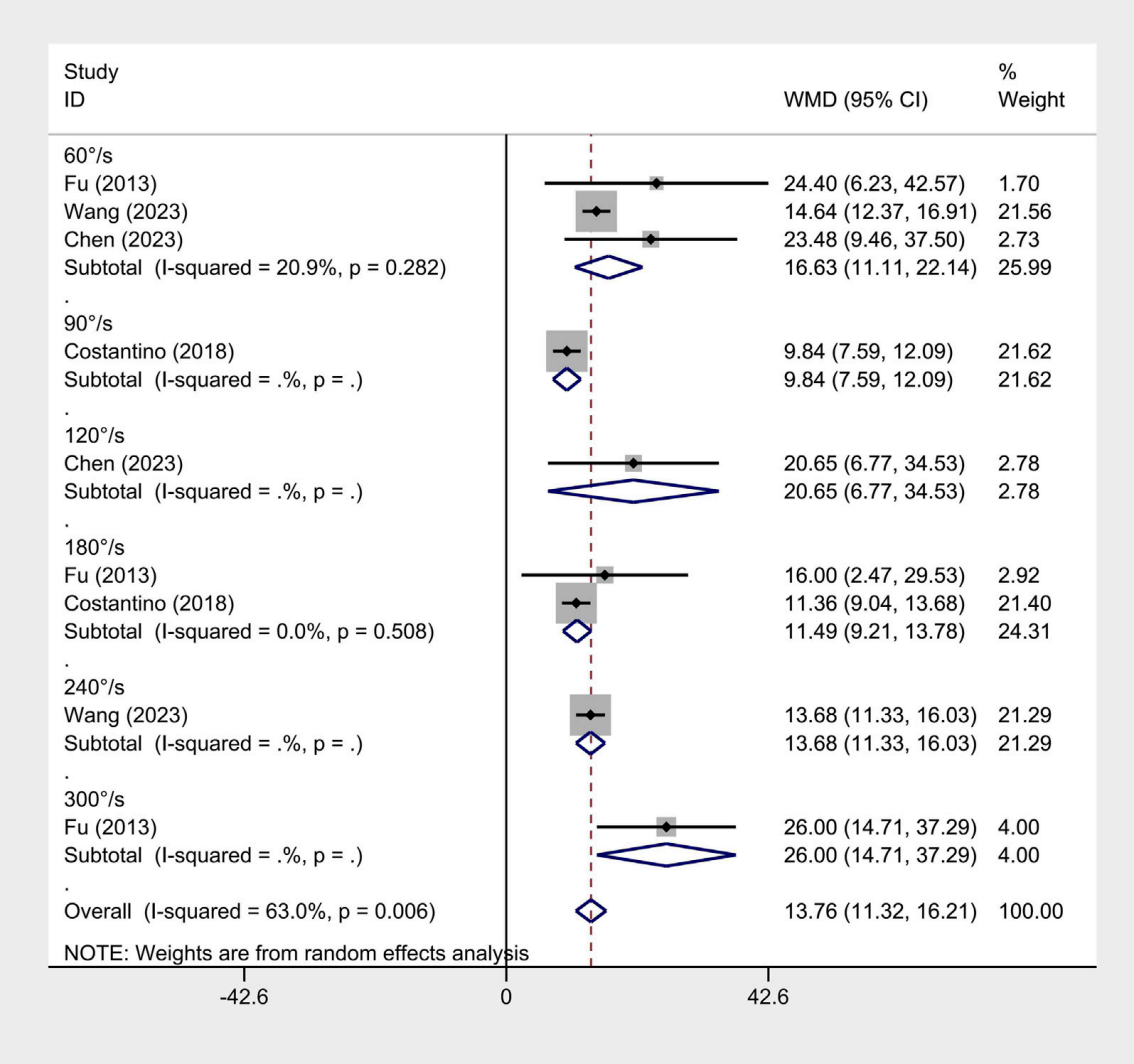
Knee joint reduction test forest map.

**FIGURE 8:**
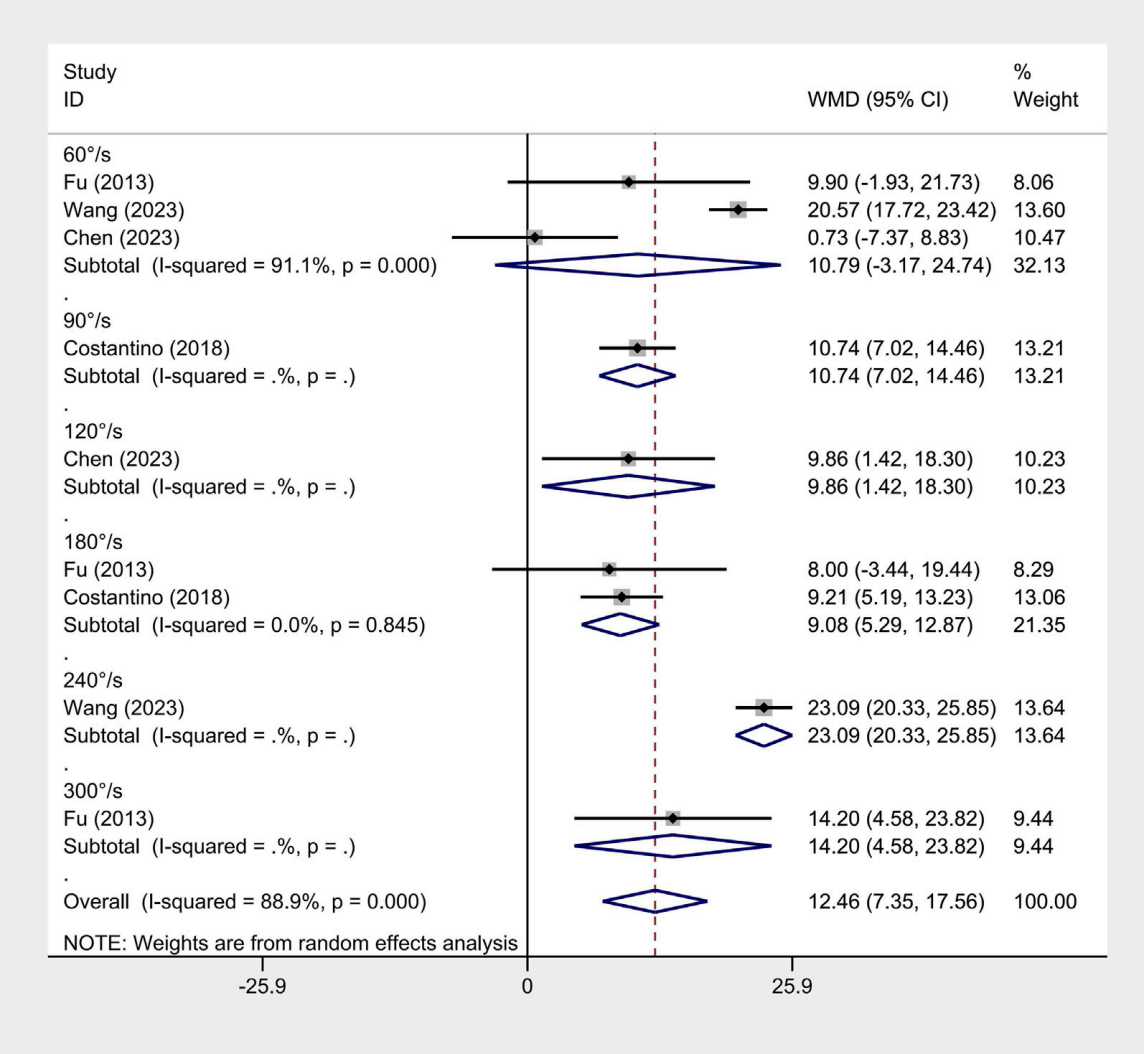
Knee stability test (OSI) forest map.

Two articles reported on the knee joint reposition test (Moezy et al., 2008; Fu et al., 2013). Due to small heterogeneity (I^2^ = 0%), a fixed-effects model was employed. Two studies applied the postural stability test (Moezy et al., 2008; Fu et al., 2013). Due to large heterogeneity (I^2^ = 77.9%), a random-effects model was utilized. ET outperformed conventional rehabilitation in both the joint reposition test (WMD = −2.01, 95% CI: −2.66 ‐ −1.37, and *p <* 0.001) and the postural stability test (WMD = −1.06, 95% CI: −1.86 ‐ −0.25, and *p* = 0.01) (Figures 7, 8). A subgroup analysis by postural stability test noted significant differences between the two groups when their eyes were closed (WMD = −1.40, 95% CI: −1.99 ‐ −0.82, and *p <* 0.001), but no difference was observed when their eyes were open (WMD = −0.82, 95% CI: −2.22 ‐ 0.59, and *p* = 0.254).

### Sensitivity analysis

Sensitivity analysis was performed for four outcomes, namely, pain score, isokinetic muscle strength, joint reposition test, and postural stability test. By individually removing one study at a time, sensitivity analysis unraveled no statistically notable changes in the meta-analysis results.

### Publication bias

Begg’s and Egger’s tests demonstrated little evidence of publication bias in the primary outcomes (VAS: Begg’s test = 1 and Egger’s test = 0.804; IFMS: Begg’s test = 0.251 and Egger’s test = 0.057; and IEMS: Begg’s test = 0.754, and Egger’s test = 0.071) or secondary outcomes (KJRT: Begg’s test = 0.707 and Egger’s test = 0.699 and OSI: Begg’s test = 0.707 and Egger’s test = 0.348) (Table 4).

**TABLE 4:** Begg’s and Egger’s test scores.

Outcomes	Begg	Egger
VAS	1	0.804
Isokinetic flexion muscle strength	0.251	0.057
Isokinetic extension muscle strength	0.754	0.071
Knee joint reposition test	0.707	0.699
Postural stability test (OSI)	0.707	0.348

VAS, visual analog scale; OSI, overall stability index.

## Discussion

This paper comprehensively compares the efficacy of the rehabilitation program with specific ET and conventional rehabilitation in patients after ACLS from 2000 to 30 August 2024. A total of 11 studies were screened, involving 552 individuals undergoing ACLS. The results evinced that the specific ET-based rehabilitation program was more effective in alleviating pain and improving joint muscle strength and knee joint functions than the conventional rehabilitation program. Subgroup analyses demonstrated marked differences in pain relief between the two rehabilitation programs in both the short and long terms, but no difference was observed in the medium term. Second, ET also enhanced the stability of the knee joint and facilitated joint reduction. Heterogeneity analyses pointed out high heterogeneity in all outcomes except for the pain and knee joint reduction test, which may be related to differences in participants and intervention durations.

Pain was assessed through the VAS scale, which determines pain levels by measuring the distance between pain-free points and marked points (Hawker et al., 2011). Our findings aligned with those reported in recent reviews on exercise interventions, which suggest that exercise can significantly reduce pain in patients (Culvenor et al., 2022; Owen et al., 2020; Fernández-Rodríguez et al., 2022). The reason for pain relief may be that exercise can improve muscle tension and modify abnormal activity patterns (Kim and Yim, 2020; Barbieri et al., 2019). This explanation is based on the perspective of muscle function. Furthermore, a recent study has suggested a link between pain relief and the regulation of the central nervous system (Belavy et al., 2021). For example, mind–body exercises such as yoga can reduce the intensity of self-perceived pain and, thus, increase pain tolerance (Cherkin and Herman, 2018). Chronic exercise training can not only induce adaptive muscle hypertrophy but also enhance the plasticity of the central nervous system, thus managing pain; vigorous exercises also reduce pain sensitivity, thereby raising the pain threshold (Belavy et al., 2021). According to the subgroup analysis, the statistical analysis revealed a significant difference in pain relief between the rehabilitation program with specific ET and conventional rehabilitation for both the short-term (1 month) and long-term (3 months) results, while the medium-term (1.5 months) results did not show any marked differences. This finding has profound implications for the timing of ET. While ensuring patients’ safety (no secondary injury or disease), early exercise rehabilitation training can relieve pain and accelerate recovery. In addition, there are differences in pain relief between different types of ET. An RCT has found that in the early postoperative period, resistance training combined with BFRT is more effective in pain relief than resistance training alone (Vieira de Melo et al., 2022). Nonetheless, our study only explored the effect of adding ET to treatment programs and failed to compare different ET types. More trials are needed to examine the differences in their efficacy.

Several articles measured muscle strength using an isokinetic muscle strength test. Despite a strict requirement for test equipment, it can well-avoid secondary injury in patients during the test. Our subgroup analysis by angular velocity proved that ET significantly improved isokinetic muscle strength more at angular velocities ranging from 60°/s to 300°/s. This result may benefit from resistance exercises (Owen et al., 2020; Fyfe et al., 2022; Hurst et al., 2022). Appropriate resistance exercise can produce certain mechanical tension, which is essential for strength recovery (Fyfe et al., 2022). Additionally, the maintenance of muscle hypertrophy requires intense stimulation. Conversely, with proper resistance, muscle strength can be increased and maintained with little need for stimulation (Fyfe et al., 2022; Chodzko-Zajko et al., 2009). Importantly, resistance exercise is deemed the only non-pharmacological treatment that can improve muscle mass and strength (Fyfe et al., 2022; Dent et al., 2018). Although the intervention duration was not analyzed, our findings showed that ET outperformed conventional rehabilitation in maintaining and increasing muscle strength in both short- and long-term studies.

In addition to pain and strength, this study also focused on knee joint function scores. The Lysholm and IKDC scsales have been used to measure knee joint function in many studies. The former is often used to evaluate knee joint function after knee ligament surgery, especially symptoms of instability (Collins et al., 2011). The latter is often used to assess knee ligament damage (Collins et al., 2011). The Lysholm scores are graded as excellent (95–100), good (84–94), and fair (65–83) (Collins et al., 2011). This study only analyzed the post-intervention Lysholm scores. After the exercise treatment, the average Lysholm score was higher than 70, and the ET group exhibited a higher overall score than the conventional rehabilitation group. Furthermore, both scales include walking, running, knee stability, and thigh atrophy (Collins et al., 2011). Frequent use of the knee joint and stress stimulation with appropriate intensity during exercise accelerated recovery and greatly improved the knee joint function. Traditionally, individuals undergoing ACLS receive passive physical therapy, which emphasizes the effectiveness of instruments with less active exercise or specific sports training. This may explain why the ET group exhibited better functional recovery.

The meta-analysis results of the knee joint reduction and postural stability tests showed that ET outperformed conventional rehabilitation. This may be ascribed to the frequent engagement of the knee joint during exercise, which can facilitate recovery, improve joint functions, and enhance the proprioception of the knee joint (Şahin et al., 2015). However, since these two outcomes are less frequently reported, supporting these findings requires the accumulation of more evidence and studies.

### Strengths and limitations

Summarizing the role of exercise interventions in ACLS patients’ rehabilitation programs, this paper represents the most recent compilation of evidence and focuses on three primary outcome measures and two secondary outcomes during knee recovery. Furthermore, compared with the current mainstream analysis of drugs and physical therapies, this study concentrates on the advantages of exercise.

There are also some limitations. Among the screened RCTs, the frequency, type, and intensity of exercise vary significantly, which may lead to some heterogeneity. Although relevant subgroup analyses were conducted for the results with high heterogeneity, the source of heterogeneity remained unclear. Furthermore, due to the insufficient data, heterogeneity was not explained comprehensively, which may introduce bias in the result reporting. Furthermore, differences in participants’ information, such as age, gender, height, weight, and type of surgery, may have affected the results. Across the studies included, the average age spanned from 18 to 29 years, and the gender ratio also varied. It is unknown whether ET is equally effective for individuals of different ages and gender. In addition, the original studies did not provide relevant information on possible adverse events, hindering further analysis in our study. Finally, because our analysis only included studies written in English, there may be some selection biases. To reduce heterogeneity, future research can include studies on the same type of exercise to investigate the effect of different frequencies, intensities, and durations of exercise.

### Practical implications

ET can help improve physical conditions in exercisers without external drug intake or physical stimulation. Therefore, such rehabilitation approaches are beneficial for patients with muscle and ligament injuries. This finding suggests that more treatment methods are available in clinical practice. For example, exercise interventions may be used instead of medications to relieve pain. Although many trials have demonstrated the benefits of ET, the appropriate type, intensity, and frequency of exercise remain to be elucidated. Meanwhile, for patients undergoing knee surgery, the timing of adding ET to rehabilitation programs remains inconclusive. Thus, a pooled analysis in this regard can provide an evidence-based basis for the formulation and updating of clinical guidelines.

## Conclusion

ET outperformed conventional rehabilitation in alleviating pain and enhancing muscle strength and knee functions in individuals undergoing ACLS. Further research is needed to investigate the appropriate frequency, intensity, and type of exercise to optimize personalized treatment for this population.

## Data Availability

The original contributions presented in the study are included in the article/Supplementary Material; further inquiries can be directed to the corresponding author.

## Abbreviations

ET, exercise therapy; ACLS, anterior cruciate ligament surgery; RCTs, randomized controlled trials; VAS, visual analog scale; ACL, anterior cruciate ligament; BPTB, bone–patella tendon–bone; OA, osteoarthritis; HT, hamstring tendon; WBVT, whole-body vibration training; BFRT, blood flow restriction training; RoB, risk of bias; MD, mean difference; WMD, weighted mean difference; CI, confidence interval; IKDC, International Knee Documentation Committee.
